# Tamoxifen and the PI3K Inhibitor: LY294002 Synergistically Induce Apoptosis and Cell Cycle Arrest in Breast Cancer MCF-7 Cells

**DOI:** 10.3390/molecules25153355

**Published:** 2020-07-24

**Authors:** Mohamed E. Abdallah, Mahmoud Zaki El-Readi, Mohammad Ahmad Althubiti, Riyad Adnan Almaimani, Amar Mohamed Ismail, Shakir Idris, Bassem Refaat, Waleed Hassan Almalki, Abdullatif Taha Babakr, Mohammed H. Mukhtar, Ashraf N. Abdalla, Omer Fadul Idris

**Affiliations:** 1 Department of Biochemistry, Faculty of Medicine, Umm Al-Qura University, Makkah 21955, Saudi Arabia; mezubier@uqu.edu.sa (M.E.A.); mathubiti@uqu.edu.sa (M.A.A.); ramaimani@uqu.edu.sa (R.A.A.); atbabakr@uqu.edu.sa (A.T.B.); mhmukhtar@uqu.edu.sa (M.H.M.); 2 Department of Biochemistry, Faculty of Pharmacy, Al-Azhar University, Assiut 71524, Egypt; 3 Department of Biochemistry and Molecular Biology, Faculty of Science and Technology, Al-Neelain University, Khartoum 11121, Sudan; amarqqqu@yahoo.com (A.M.I.); ibnomer57@yahoo.com (O.F.I.); 4 Department of Laboratory Medicine, Faculty of Applied Medical Sciences, Umm Al-Qura University, Makkah 7607, Saudi Arabia; siidris@uqu.edu.sa (S.I.); barefaat@uqu.edu.sa (B.R.); 5 Department of Pharmacology and Toxicology, Faculty of Pharmacy, Umm Al-Qura University, Makkah 21955, Saudi Arabia; whmalki@uqu.edu.sa; 6 Department of Pharmacology and Toxicology, Medicinal and Aromatic Plants Research Institute, National Center for Research, Khartoum 2404, Sudan

**Keywords:** breast cancer, tamoxifen, LY294002, synergism, apoptosis, cell cycle

## Abstract

Breast cancer is considered as one of the most aggressive types of cancer. Acquired therapeutic resistance is the major cause of chemotherapy failure in breast cancer patients. To overcome this resistance and to improve the efficacy of treatment, drug combination is employed as a promising approach for this purpose. The synergistic cytotoxic, apoptosis inducing, and cell cycle effects of the combination of LY294002 (LY), a phosphatidylinositide-3-kinase (PI3K) inhibitor, with the traditional cytotoxic anti-estrogen drug tamoxifen (TAM) in breast cancer cells (MCF-7) were investigated. LY and TAM exhibited potent cytotoxic effect on MCF-7 cells with IC_50_ values 0.87 µM and 1.02 µM. The combination of non-toxic concentration of LY and TAM showed highly significant synergistic interaction as observed from isobologram (IC_50_: 0.17 µM, combination index: 0.18, colony formation: 9.01%) compared to untreated control. The percentage of early/late apoptosis significantly increased after treatment of MCF-7 cells with LY and TAM combination: 40.3%/28.3% (*p* < 0.001), compared to LY single treatment (19.8%/11.4%) and TAM single treatment (32.4%/5.9%). In addition, LY and TAM combination induced the apoptotic genes Caspase-3, Caspase-7, and p53, as well as p21 as cell cycle promotor, and significantly downregulated the anti-apoptotic genes Bcl-2 and survivin. The cell cycle assay revealed that the combination induced apoptosis by increasing the pre-G_1_: 28.3% compared to 1.6% of control. pAKT and Cyclin D1 protein expressions were significantly more downregulated by the combination treatment compared to the single drug treatment. The results suggested that the synergistic cytotoxic effect of LY and TAM is achieved by the induction of apoptosis and cell cycle arrest through cyclin D1, pAKT, caspases, and Bcl-2 signaling pathways.

## 1. Introduction

Worldwide, breast cancer (BC) has the highest incidence rate (24.2%) of all cancers in women with more than two million newly diagnosed cases and almost 627,000 deaths (15%) occurred in 2018 [1]. In Saudi Arabia, BC has the largest number of incidences between females (3629 cases: 29.7% of total malignancies) [2]. Nevertheless, frequent tumor recurrence results in poor prognosis of BC patient of which less than 5% survive for more than ten years [3]. Discovering new therapies with improved pharmacokinetics is therefore required for improving the outcome of BC treatment [4]. 

BC is classified according to the gene expression of estrogen receptor (ER) and human epidermal growth factor receptor 2 (HER2) into five major molecular subtypes, which are different in growth and prognosis. Theses subtypes include luminal A (ER^+^/HER2^−^/low levels of Ki-67 protein), luminal B (ER^+^/HER2^−/+^/high levels of Ki-67 protein), triple-negative/basal-like (ER^−^/HER2^−^), HER2 enriched (HR^−^/HER2^+^), and normal-like BC, which is similar to luminal A, but with poor prognosis [5]. Luminal A and luminal B breast cancer are the most dominant subtype, affecting more than 73% of total BC patients [6].

Tamoxifen (TAM) is the oldest and most-prescribed selective estrogen receptor modulator, that has been approved to treat women and men diagnosed with hormonal receptor (HR^+^), early-stage BC after surgery to reduce the risk of the cancer recurrence as well as treatment of advanced-stage or metastatic HR^+^ BC patients [7,8,9]. The combination of high efficacy in both pre- and postmenopausal women and a good tolerability profile of TAM lead to maintain its position as drug of choice for most patients with HR^+^ breast cancer [7]. In addition, TAM has been used as chemopreventive agent to reduce breast cancer risk in women, who haven’t been diagnosed, but are at higher-than-average risk for incidence of BC [10]. These high therapeutic benefits of TAM by binding with the ER causing apoptotic effect on the mammary cells [7,11]. 

The development of both de novo and acquired resistance to TAM is a significant problem. Recent advances in our understanding of the molecular mechanisms that contribute to resistance have provided means to predict patient responses to TAM and develop rational approaches for combining therapeutic agents with TAM to avoid or desensitize the resistant phenotype [12]. Overcoming the anti ER drug resistance can be achieved by the introduction of new drug classes and combinations that can synergistically improve the efficacy and decrease the effective dose, hence decrease the side effects. Long term estrogen deprivation (LTED) treatment among ER+ BC cells results in adaptive increase in ER expression, which is followed by activation of multiple tyrosine kinases. Combination therapy with the ER down-regulator fulvestrant and the broad kinase inhibitor dasatinib exhibited synergistic activity against LTED cells, by a reduction of cell proliferation, survival, and invasion [13].

In addition, the phosphatidylinositide-3-kinase (PI3K)/Akt signaling pathway is considered as the ideal pathway to explain the transmission of anti-apoptotic signals for cancer cell survival and regulate cell growth, proliferation, transcription, and metabolic processes [14]. The activation of PI3K/Akt signaling pathway is associated with poor prognosis in BC [14]. Inhibitors of PI3K/Akt have undergone pre-clinical evaluation with encouraging results and considered to be one of the most promising targeted therapies for cancer treatment [15]. LY294002 (LY) is a morpholine containing compound with potent inhibitory action for numerous proteins, and a strong inhibition of PI3Ks, which causes induction of apoptosis in tumor cells, but the precise mechanism of its antitumor activity is not completely well understood, as it was also shown to inhibit the invasiveness of cancer cells by downregulating the expression of MMP-2, MMP-9, and VEGF, and reducing MVD [16]. LY, among other PI3K inhibitors, did not reach the clinical trials phase because of its weak drug ability and toxicity [17]. 

This study was conducted to show the effects of LY or TAM alone or in combination against MCF-7 (ER^+^) cells. The underlying mechanisms of the possible synergistic effects of this combination are further explored in order to develop a novel and effective therapeutic combination against BC and to reduce the toxicity and resistance of LY and TAM.

## 2. Results

### 2.1. LY294002 and Tamoxifen Synergistically Inhibited Breast Cancer Cells Proliferation

**Figure 1:**
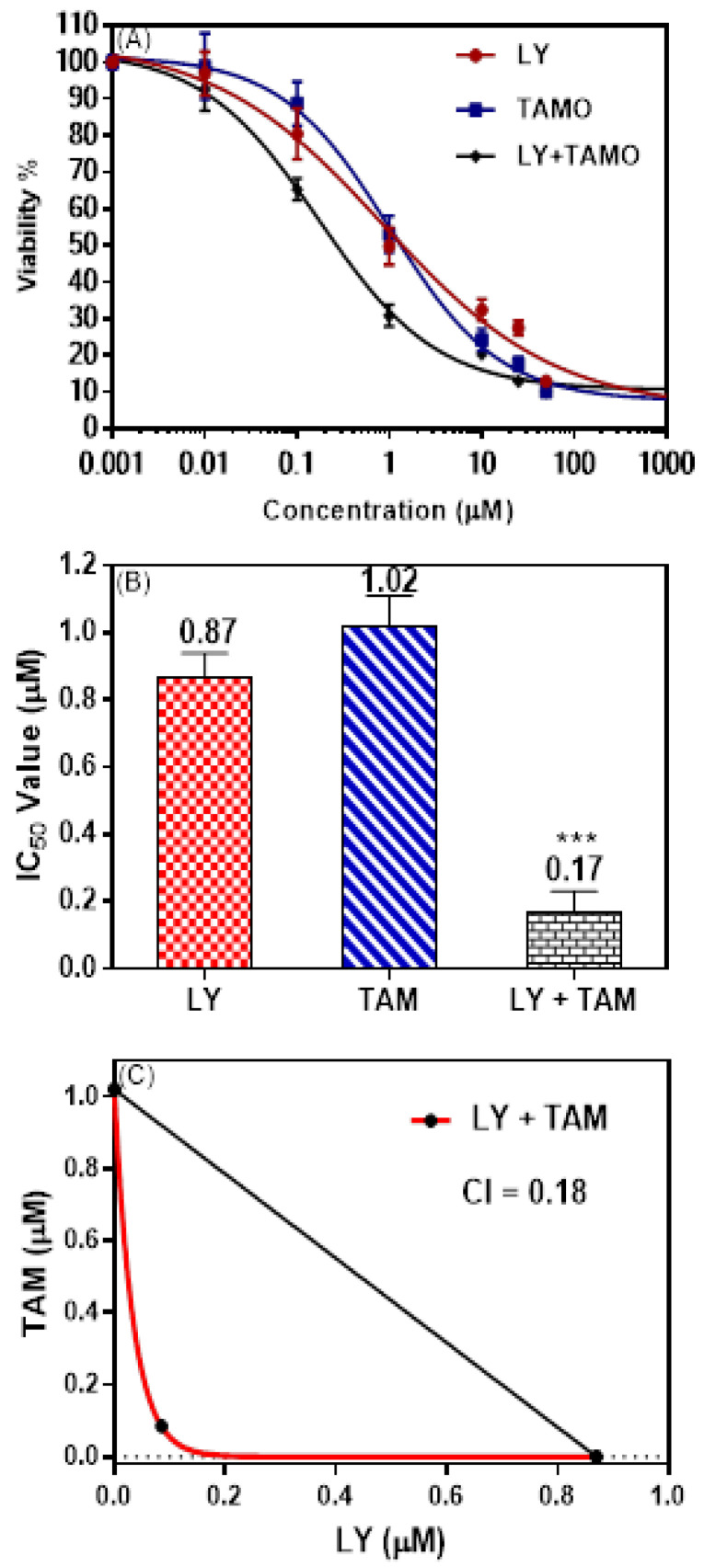
(**A**): Dose response curve, (**B**): IC_50_ values, (**C**): isobolgram and combination index of MCF-7 cells treated with different concentrations of LY, TAM, and LY + TAM combination for 72 h. The IC_50_ was calculated using GraphPad Prism V6 by fitting of sigmodal four parameter curve. The data expressed as mean ± SD (*n* = 3, of three experiments). Statistical differences, compared with the control cells, were assessed by a one-way ANOVA with the Tukey’s post-hoc multiple comparison test (GraphPad Prism). *p* < 0.001 (***) was taken as significant.

**Figure 2:**
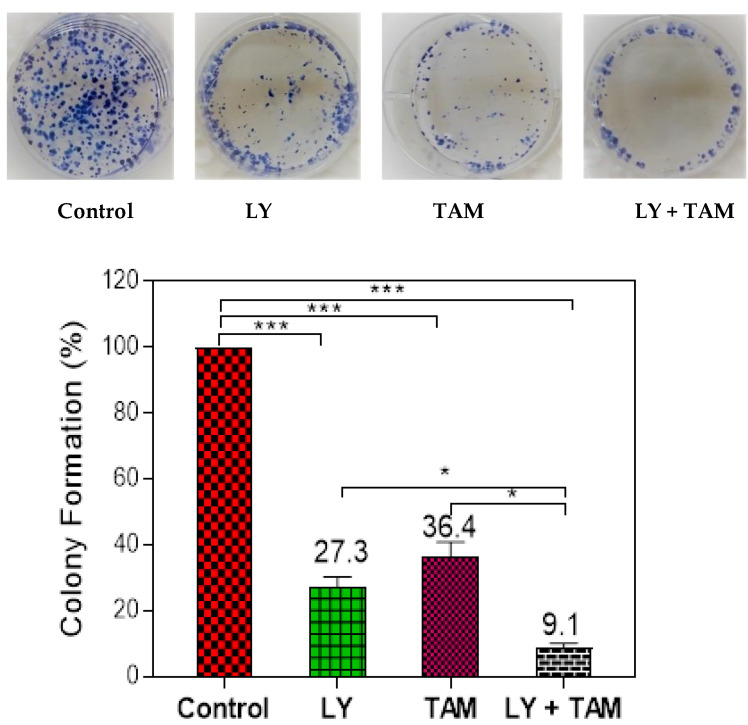
Colony formation assay of MCF-7 cells treated with LY, TAM, and LY + TAM combination. MCF-7 cells were treated for 24 h with the experimental set and cells were seeded in 6-well plates (200 cells/well) and incubated for 14 days. The colonies were counted after staining with methylene blue. The colony formation of the treatment set was quantified as a percentage related to untreated control. Statistical differences, compared with the control cells, were assessed by a one-way ANOVA with the Tukey’s post-hoc multiple comparison test (GraphPad Prism).). *p* < 0.05 (*), *p* < 0.001 (***) was taken as significant.

The ability of LY to improve the cytotoxicity of tamoxifen on MCF-7 breast cancer was evaluated using an MTT assay. Figure 1A shows dose-response curve of MTT assay. The down deviation of curve was observed in MCF-7 cell treated with LY + TAM combination comparing to each of LY or TAM alone. The combination showed significant synergistic interaction with decrease in IC_50_ in MCF-7 cells (0.17 µM) comparing to LY (0.87 µM) and TAM (1.02 µM) treated cells (Figure 1B). Non-toxic concentration 100 nM (85% live cells) from LY and TAM was selected to use in all experimental sets. The synergistic effect of the combination was elucidated from isobologram and combination index value (0.18) Figure 1C. To confirm the synergistic interaction of LY and TAM, the plate colony formation assay was performed. As shown in Figure 2, the combination of LY and TAM exhibited a significant lower percentage of colony formation (9.1%) compared to the control, where LY treated cells showed (27.3%) and TAM showed (36.4%) compared to the control MCF-7 cells. A non-significant difference between LY and TAM treated cells was observed. 

### 2.2. LY294002 and Tamoxifen Induced Apoptosis in Breast Cancer Cells

**Figure 3:**
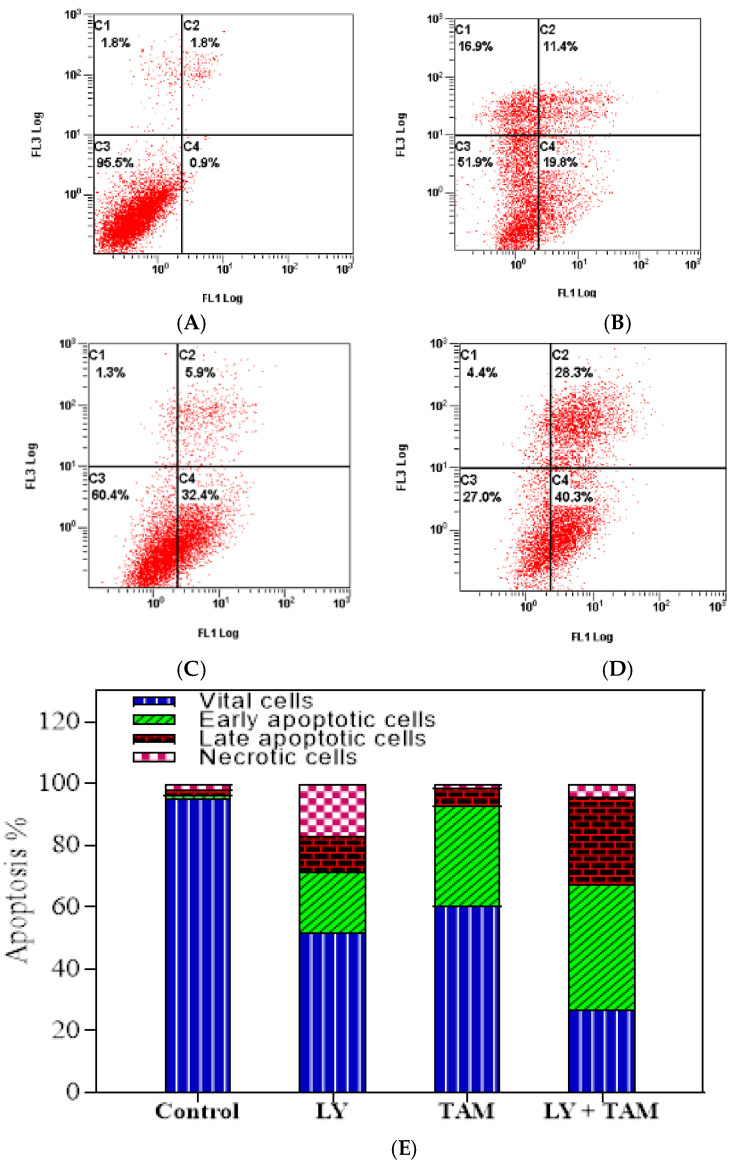
The induction of apoptosis in MCF-7 cells treated with (**A**): control, (**B**): LY, (**C**): TAM, and (**D**): LY + TAM combination for 24 h. Followed by Annexin V FITC/PI staining. The scattered plot *X* axis: FL1 for Annexin V, *Y* axis: FL3 for PI. (**E**): Columns represent the flow cytometry data analysis as means of the percentages of vital, early apoptotic, late apoptotic, and narcotic cells (*n* = 3 of three independent experiments).

**Figure 4:**
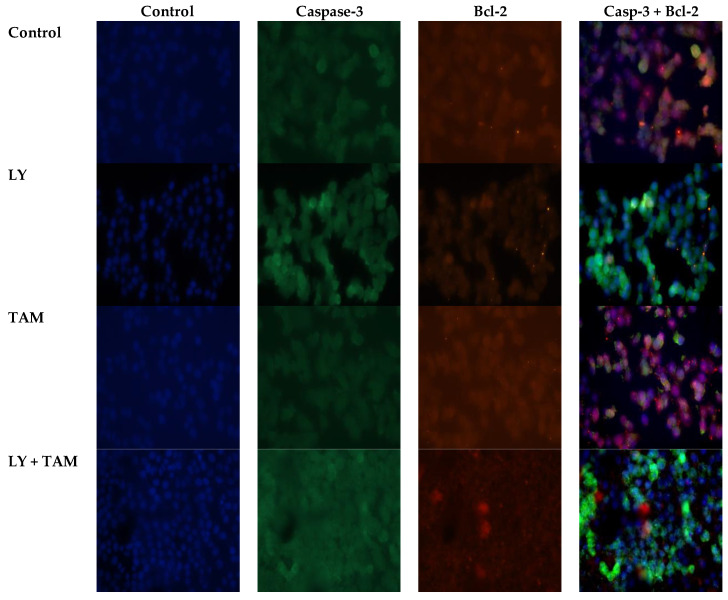
The induction of apoptosis in MCF-7 cells treated with LY, TAM, and LY + TAM combination 24 h. Images taken with confocal microscope (EVOS FL, scale bar 20 nM) to evaluate the expression of apoptotic (Caspase-3) and antiapoptotic (Bcl-2) markers. The images show green and red color staining for Caspase-3 and Bcl-2, respectively. Overlay images represent the fluorescence intensity of both apoptotic markers.

**Figure 5:**
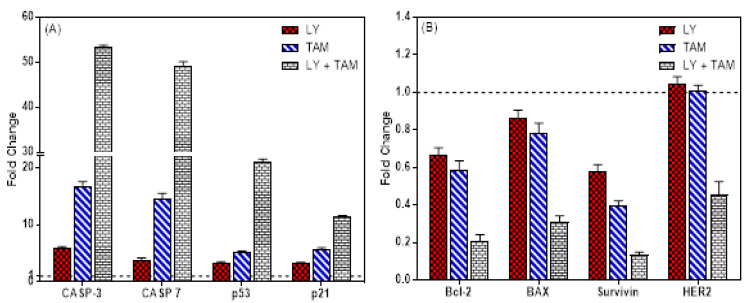
The expression of apoptosis genes in MCF-7 cell after treatment with LY, TAM, and LY+ TAM combination for 24 h. The total RNA was extracted and the mRNA levels of upregulated genes (**A**) and downregulated genes (**B**) was quantified using RT-PCR. The data represented the mean of the fold change related to untreated control (fold change = 1 dashed line).

In order to elucidate the underlying mechanism of the synergistic inhibition of BC cell growth by LY and TAM combination, apoptosis analysis was performed through annexin V FITC/PI double staining. The data revealed that each of LY and TAM were able to induce early/late apoptosis 19.8%/11.4% and 32.4/5.9%, respectively (Figure 3). However, the combination of LY with TAM significantly increased the early/late apoptosis to 40.3/28.3% (*p* < 0.001). To explore the molecular mechanism of increasing in the apoptotic MCF-7 cells, anti-apoptotic and apoptotic genes were measured by immunofluorescence in MCF-7 cells. As shown in Figure 4, the treatment of MCF-7 cells by LY + TAM increased the expression of Caspase-3 and decreased the expression of Bcl-2 compared to the cells treated with either LY or TAM alone. In addition, Figure 5A shows that LY +TAM significantly increased the expression of Caspase-3 3.2 and 9.2-times more compared to TAM and LY alone, respectively. Moreover, caspase-7 was overexpressed in MCF-7 cells 3.4 and 12.6 times higher in treated cells with LY +TAM compared to cells treated with TAM and LY single treatment, respectively. The combination also significantly induced the expression of both p53 and p21: 4 and 2 times more compared to LY, and 6.3 and 3.6 times more compared to TAM, respectively. Additionally, the combination decreased the Bcl-2, BAX, and survivin 2.8 times, 2.5 times, and 3 times more than single treatment with TAM, and 3.1 times, 2.8 times, and 4.46 times more than single treatment LY, respectively. Finally, LY and TAM did not exhibit any change in HER-2 gene, while the combination decreased the expression of HER-2 to 0.45 folds compared to untreated control (Figure 5B)

### 2.3. LY294002 and Tamoxifen Induced Cell Cycle Arrest in Breast Cancer Cells

**Figure 6:**
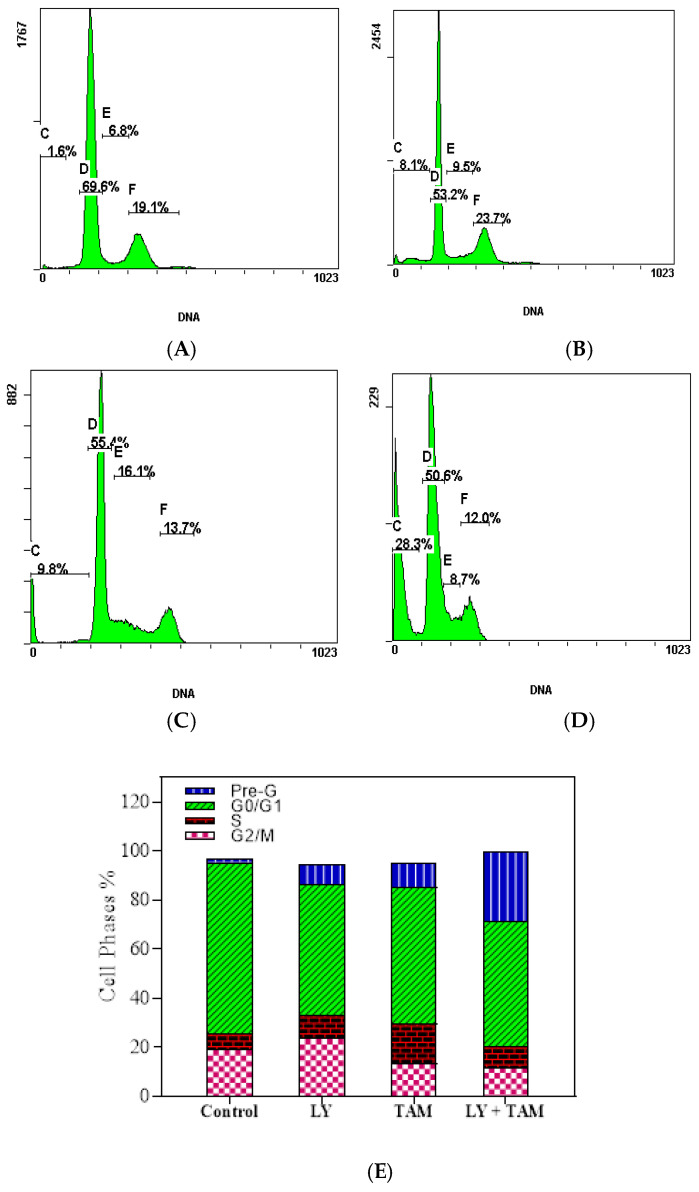
Histograms represent DNA cell cycle distribution in MCF-7 cells treated with (**A**): control, (**B**): LY, (**C**): TAM, and (**D**): LY + TAM combination for 24 h. (**E**): columns represent the flow cytometry data analysis as means of the percentages of pre-G_1_, G_0_/G_1_, S, and G_2_/M (*n* = 3, three independent experiments).

The effect of LY, TAM, and LY + TAM combination on the DNA content of MCF-7 cells was assessed using PI staining. The treatments lead to a significant increase in the apoptotic pre-G1cell population phase from 1.6% (untreated control) to 8.1%, 9.8%, and 28.3% in the treated cells with LY, TAM, and LY + TAM, respectively as shown in Figure 6. The cell population of G_0_/G_1_ phase decreased from 69.6% to 53.2%, 55.4%, and 50.6% after treatment with LY, TAM, and their combination, respectively. A non-significant decrease in S phase cell population was observed in all experimental set compared to untreated cells (6.8%). The G_2_/M cell population was reduced in cells treated with LY (23.7%), TAM (13.7%, *p* < 0.05), and combination (12%, *p* < 0.05) compared to untreated cells (19.1%) (Figure 6).

### 2.4. pAKT and Cyclin D1 Decreased in MCF-7 Cells Treated with LY294002 and Tamoxifen

**Figure 7:**
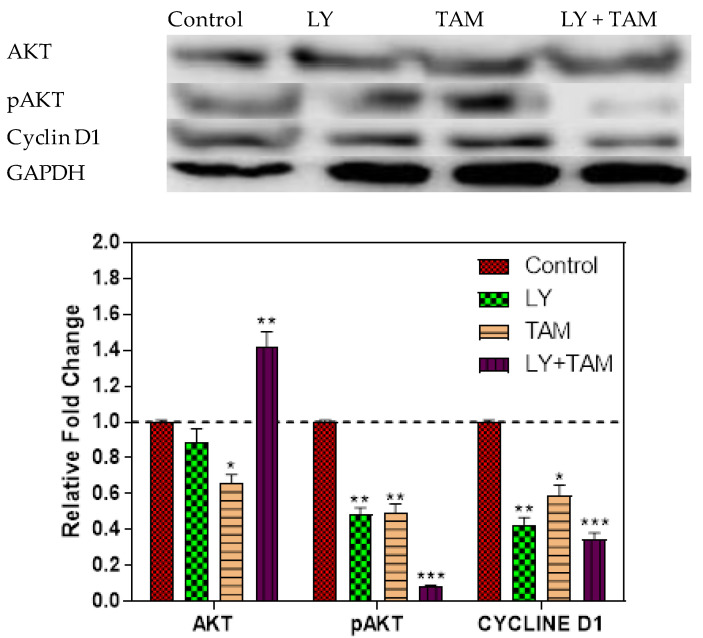
MCF-7 cells were incubated with control, LY, TAM, and LY + TAM for 24 h. Proteins from total cell lysate were separated by SDS-PAGE gel electrophoresis, and immunoblotted with antibodies against AKT, phosphorylated AKT, and cyclin D1. The color density was quantified using densitometry). Statistical differences, compared with the control cells, were assessed by a one-way ANOVA with the Tukey’s post-hoc multiple comparison test (GraphPad Prism). *p* < 0.05 (*), *p* < 0.01 (**) and *p* < 0.001 (***) were taken as significant.

To explore the molecular targeting of PI3K signaling, AKT, pAKT, and cyclin D1 were assessed after 24 h of the treatment with combination. A Significant decrease of pAKT and cyclin D1 was seen after treatment with LY, ATM, and LY + TAM combination compared to untreated control (Figure 7).

## 3. Discussion

The phosphatidylinositide-3-kinase (PI3K)/Akt signaling pathway regulates many biological processes including cancer cell growth and metastasis [18,19]. Consequently, aberrant activation of the PI3K/Akt signaling pathway is frequently associated with progressive BC, which could be resistant to anticancer therapies [20]. It has been estimated that upregulation of PI3K signalling is involved in around 70% of BC cases [21]. Several PI3K/Akt signaling inhibitors have been effective in inhibiting progression of tumors during pre-clinical and clinical trials and approved by United States Food and Drug Administration (FDA) [22,23]. However, most of these inhibitors have demonstrated only modest clinical efficacy as monotherapies in BC because of drawbacks in their pharmacokinetics and tolerability [23]. Therefore, the combination of PI3K/Akt signaling inhibitors with radiation or chemotherapy is considered as a dynamic research area, approached to overcome therapeutic resistance and enhance treatment efficacy [24]. In a previous study, the tumor associated macrophages were shown to accelerate the endocrine resistance of MCF-7 cells treated with TAM, due to activation of the PI3K/Akt/mTOR signaling pathway [25]. Thus, the co-targeting of ER and PI3K/Akt pathway may stand as a new therapeutic target.

This study was conducted to evaluate the possible synergistic cytotoxic combination effect of LY: as a specific phosphatidylinositide-3-kinase (PI3K) inhibitor, and TAM: as an established BC/ER^+^ drug. MCF-7 cells were used as a model of ER^+^ BC. Our results uncovered that the non-toxic dose of LY and TAM synergistically enhanced their cytotoxicity and clonogenecity against MCF-7 with a significant decrease in IC_50_ and combination index (Figure 1 and Figure 2). The cytotoxicity of LY and TAM as single treatments and in combination were previously evaluated on A2780 (ovarian cancer) and MRC-5 (normal fibroblast). The IC_50_ of LY were 21.2 µM and 35.7 µM, and for TAM were 10.4 µM and 11.4 µM, and for the combination of LY and TAM were 4.7 and 24.2 µM, respectively [26,27,28]. When that result was compared with the result of this study, LY and TAM were found to be more effective in MCF-7 cells compared to A2780 cells. In other previous studies, the co-treatment of LY and TAM significantly enhanced the cytotoxicity against lung and brain cancer cells compared to treatment with TAM alone [27,29].

In the second part of this study, the underlying mechanism of the synergistic apoptosis-inducing effect resulting from the treatment of MCF-7 cells with LY and TAM combination, could be explained by the increase of released apoptotic molecules or the decrease of released anti-apoptotic ones. Some of these key apoptotic molecules, which are used as indicators of apoptosis in BC are caspase-3, -7, and p53 [30,31,32,33] and p21 as cell cycle promotors [34]. In addition to the Bcl-2 family, which are also considered as important anti-apoptotic genes, their overexpression is frequently related to cancer development [35]. The LY and TAM combination decreased the expression of Bcl-2 and increased the expression of caspase-3 in MCF-7 cells as observed from immunofluorescence experiment (Figure 4). These data were confirmed by determination of mRNA levels of apoptosis genes Bcl-2, BAX, surviving, HER2, p53, p21, caspase-3, and caspse-7 after treatment of MCF-7 cells with LY, TAM, and their combination (Figure 5). 

The downregulation of Bcl-2 and survivin was more significant by treatment with the combination more compared to single treatments, which may partly explain the weaker effect of LY or TAM on the induction of apoptosis. While, the non-significant downregulation of BAX might be explained by indirect interaction of LY, TAM or combination with BID/BIM, which required to initiate membrane permeabilization and apoptosis [36]. Survivin is a pro-survival gene and its overexpression is observed in most cancers. It is associated with resistance to chemotherapy and radiation, thus possibly leading to the failure of therapy and poor prognosis [37,38]. Therefore, through the downregulation of anti-apoptotic genes (Bcl-2 and surviving) and overexpression of apoptotic genes (p53 and caspases) the resistance against TAM in breast cancer cells can be reversed.

In this study, the treatment with LY, TAM, and their combination increased the expression of caspase-3 in MCF-7 cells. There are debate in the expression of caspase-3 in MCF-7. Several studies measured levels of caspase-3 indirectly via fluorometric assay systems and by western blotting analyses and reported that directly the presence of this protease in MCF-7 cells [39,40]. However, other reports stated that MCF-7 cells do not express caspase-3 [41]. Our results supposed that the expression of caspase-3 is deficient in untreated MCF-7 cells, but by treatment with LY, TAM, and their combination, the expression of genes was increased as an indication of apoptosis induction by their treatment. 

In this study, the induction of apoptosis by LY, TAM, and their combination was confirmed by annexin V/PI double staining using flow cytometry (Figure 3). The pattern of cell death in LY treated cells was suggest necrosis rather than apoptosis Figure 3B. The LY dose (100 nM) that was used in this experiment might be too high to induce necrosis. Previously, it has been reported that LY induced apoptosis and necrosis in mice depending on the treated dose [42].

The combination of LY and TAM increased the percentage of cells in pre-G_1_ cell cycle phase and decreased the percentage of cells in G_0_/G_1_, S and G_2_/M phases compared to untreated cells (Figure 6), thus indicating the down regulation of the cell cycle regulation genes. It has been previously reported that one of major problems in BC is the occurrence of cross-resistance that develops due to the change in the expression of DNA damage repair or cell cycle genes [43]. The phosphorylation of AKT can mediate BC resistance to therapy [44,45]. We have shown in this study a significant decrease in the levels of pAKT and cyclin D1 after treatment with LY and TAM combination better than the decreasing effect of LY or TAM alone (Figure 7). These results indicated that the downregulation of the pAKT signaling pathway could also be responsible for the synergistic cytotoxic effect of LY and TAM combination in MCF-7.

## 4. Materials and Methods

### 4.1. Compounds and Reagents

LY294002 and tamoxifen were purchased from Selleckchem, Houston, TX, USA. All reagents and kits used in this study were purchased from Sigma-USA, unless other manufacturer is mentioned. 

### 4.2. Cell Culture

MCF-7 cells (breast adenocarcinoma) was obtained from the ATCC. For sub-culture, RPMI-1640 media (10% FBS; 1% Antibiotic-Antimycotic, Gibco) was used. Cells were kept at 95% humidity, 37 °C, and 5% CO_2_ for up to 10 passages. Mycoplasma was tested monthly using the bio-luminescence kit (Lonza, Visp, witzerland) and read by a multi-plate reader.

### 4.3. Cytotoxicity and Combination Studies

MTT assay was used for evaluation the cytotoxic effects of LY and TAM and their combination according to previous reports [46,47]. MCF-7 cells were cultured in 96-well (1 × 10^3^/well). Cells were treated with several concentrations (0.001–50 µM) of LY and TAM. Cells were incubated for 72 h, followed by addition of MTT for 3 h (Life technologies). The formazan crystals were dissolved in DMSO (100 µL) and the light absorbance was measured used BIORAD PR 4100 microplate reader at λmax 570 nm. IC_50_ were calculated using GraphPad Prism. Nontoxic concentrations (~80% of viable cells) from LY (100 nM) and TAM (100 nM) were used for all combination experiments of this study. 

### 4.4. Clonogenic Survival Assay

The cytotoxicity of LY, TAM and the synergistic cytotoxic effect of their combination was confirmed by clonogenic survival assay according to previous report [48]. Briefly, low density MCF-7 cells (2 × 10^2^ cells/well) were cultivated in 2 mL media in 6-well plates in duplicates. Plates were incubated at 37 °C overnight to allow attachment. Cells were treated with either LY (100 nM), TAM (100 nM) or their combinations. Plates were incubated at 37 °C for 24 h, then medium containing compounds were aspirated, and replaced with 2 mL fresh media. Plates were checked under the microscope every 2 days, and cells forming a colony were counted. After 14 days, colonies which containing at least 50 cells were counted. Following the aspiration of media, cells were washed with cold PBS, then fixed with cold methanol for 5 min at room temperature. Cells were stained with 0.5% *v*/*v* methylene blue in methanol: H_2_O (1:1) for 15 min. Colonies were washed with PBS and H_2_O. Plates were left to dry, before counting colonies.

### 4.5. Apoptosis Assay Using Flow Cytometric Analysis

The ability of LY, TAM and their combinations to induce apoptosis in BC cells was quantified by flow cytometry using annexin V FITC/PI (propidium iodide) double staining assay following previous report [49,50]. MCF-7 cells (5 × 10^5^ cells/well) were cultivated in 6 well plates for 24 h. LY (100 nM), TAM (100 nM), and their combinations were incubated with cells for further 24 h, before harvested cells were labeled by annexin V FITC/PI apoptosis detection kit (Invitrogen) according to the manufacturer’s instruction. Apoptotic cells (early and late) were quantified as % by flow cytometer (FC500, Beckman Coulter, Miami, FL, USA). 

### 4.6. Immunofluorescence Staining 

The induction of apoptosis in MCF-7 cells by LY, TAM, and their combinations were confirmed by immunofluorescence staining assay to determine the co-localization of the antiapoptotic marker (Bcl-2) and the apoptotic marker (Caspase-3). MCF-7 cells were treated with LY, TAM, and their combinations for 24 h. MCF-7 cells (5 × 10^3^/chamber) were seeded and incubated for 24 h. Then cells were blocked with normal donkey serum (30 min), followed by incubation with the primary mouse monoclonal and rabbit polyclonal IgG antibodies (1:200, 3 h) (Santa Cruz Biotechnology, Inc., Santa Cruz, CA, USA) for the detection of Bcl-2 and Caspase-3, respectively. Then, slides were incubated with a mixture of tagged cross-adsorbed donkey anti-mouse (Alexa Fluor 488) and anti-rabbit (Alexa Fluor 555) IgG secondary antibodies (Thermo Fisher Scientific) for 60 min. Slide sections were counterstained with ProLong Diamond Anti-fade Mountant including 4′,6-diamidino-2-phenylindole (DAPI; Thermo Fisher Scientific, Waltham, MA, USA). EVOS FL microscopy (Thermo Fisher Scientific, Waltham, MA, USA) was used for slide examination. Digital images were taken with 40× objective.

### 4.7. Quantitative Real-Time PCR

For more elucidation of the apoptotic effects of LY, TAM, and their combinations in BC, the RT-PCR platform (Applied Biosystems 7500 Fast Real Time PCR System, Waltham, MA, USA) was applied. RT-PCR was used to quantify the expression of the apoptosis genes: caspase-3, caspse-7, p53, p21, Bcl-2, BAX, Survivin, and Her2 in MCF-7 cells [51]. Briefly, MCF-7 cells (1 × 10^6^ cells/well) were cultivated in 6 well plates for 24 h, then cells were treated with LY, TAM, and their combinations for 24 h. Total RNA was isolated according to manufactory instruction. The RT-PCR experiment was conducted with a mixture of cDNA, 2X SYBR Green I Master mix, PCR-grade water, forward and reversed human primers of selective genes, and GAPDH as housekeeping gene (Applied-Biosystems, Thermo Fisher Scientific, Waltham, MA, USA) (Table 1). 

### 4.8. Cell Cycle Analysis 

Cell cycle analysis was applied to explore the underlying mechanisms of cytotoxic effects of LY, TAM, and their combinations in BC. MCF-7 cells (5 × 10^5^ cells/well) were treated with LY, TAM, and their combinations for 24 h. Cells were then fixed in 70% ethanol and processed for cell cycle analysis, after staining with propidium iodide (PI, Santa Cruz). FC500, Beckman Coulter, Miami, FL, USA flow cytometer was used for analyzing a total of 20,000 single-cells, with the aid of Expo 32 software, Miami, FL, USA [52].

### 4.9. Western Immunoblotting

Identification of the expression change of cell cycle proteins (AKT, pAKT, CyclinD1, and GAPDH) was confirmed by immunoblotting assay. MCF7 cells (1 × 10^6^ cells/well of 6 well plate) were treated with LY, TAM, and their combinations for 24 h. Lysis buffer was used to isolate total proteins. The Bradford Method was used to determine the concentration of total proteins, which were electrophoresed using a polyacrylamide gel and transferred to membrane. The membrane was incubated with AKT, pAKT cyclin D1 antibodies (Cell signalling) for 2 h at room temperature and secondary antibody GAPDH for 1 h. Horseradish peroxidase (HRP)-conjugated secondary antibodies were used to visualize the immunoreactivity by chemiluminescence, and images were captured by a scanner (GeneGenome, Syngene Bioimaging, Cambridge, CB4 1TF, United Kingdom) [51].

### 4.10. Statistics

Statistical differences were assessed by one-way ANOVA with the Tukey’s post-hoc multiple comparison test. *p* < 0.05 (*), *p* < 0.01 (**), *p* < 0.001 (***), and *p* < 0.0001 (****) were taken as significant.

## 5. Conclusions

Our results demonstrated that the synergistic cytotoxic effect of LY and TAM is achieved by the induction of apoptosis and cell cycle distribution through cyclin D1, pAKT, caspases, and Bcl-2 signaling pathways, all which might help in reversing the resistance of MCF-7 cells to TAM and decrease the toxicity of LY. Further in vivo and genetic studies are needed to explore more information about the efficacy and molecular targeting of this combination. 

## Figures and Tables

**Table 1:** Sequence of primers used in RT-PCR.

Gene	Sequence
**Caspase-3**	F: ACATGGAAGCGAATCAATGGACTC R: AAGGACTCAAATTCTGTTGCCACC
**Caspase-7**	F: GGACCGAGTGCCCACTTATC R: TCGCTTTGTCGAAGTTCTTGTT
**p53**	F: CCA CCA TAA AGC TGG GGC TT R: TCT CCC CGC CTC TTT GAC TC
**p21**	F: GAG TCC TGT TTG CTT CTG GGC A R: CTG CAT TGG GGC TGC CTA TGT A
**BCL-2**	F: CTCTCGTCGCTACCGTCGCG R: AGGCATCCCAGCCTCCGTTATCC
**BAX**	F: GCCCTTTTGCTTCAGGGTTT R: TCCAATGTCCAGCCCATGAT
**Survivin**	F: TTGCTCCTGCACCCCAGAGC R: AGGCTCAGCGTAAGGCAGCC
**HER2**	F: CCT CTG ACG TCC ATC GTC TC R: CGG ATC TTC TGC TGC CGT CG
**GAPDH**	F: AGGTCGGTGTGAACGGATTTG R: TGTAGACCATGTAGTTGAGGTCA

The RT- PCR program was run in 41 cycles of denaturation at 95 °C for 15 s followed by annealing/extension at 60 °C for 60 s (*n* = 3). Standard comparative method was used to evaluate the genes expression, where the raw Ct values were converted into relative expression levels (fold change: 2^−∆∆*C*t^).

## References

[1] Bray F., Ferlay J., Soerjomataram I., Siegel R.L., Torre L.A., Jemal A. (2018). Global cancer statistics 2018: GLOBOCAN estimates of incidence and mortality worldwide for 36 cancers in 185 countries. CA Cancer J. Clin..

[2] Al-Shahrani Z., Al-Rawaji A.I., Al-Madouj A.N., Hayder M.S. (2017). Cancer Incidence Report Saudi Arabia 2014.

[3] Sopik V., Sun P., Narod S.A. (2019). Predictors of time to death after distant recurrence in breast cancer patients. Breast Cancer Res. Treat..

[4] Alkahtani H.M., Alanazi M.M., Aleanizy F.S., Alqahtani F.Y., Alhoshani A., Alanazi F.E., Almehizia A.A., Abdalla A.N., Alanazi M.G., El-Azab A.S. (2019). Synthesis, anticancer, apoptosis-inducing activities and EGFR and VEGFR2 assay mechanistic studies of 5,5-diphenylimidazolidine-2,4-dione derivatives: Molecular docking studies. Saudi Pharm. J..

[5] Abubakar M., Sung H., Devi R., Guida J., Tang T.S., Pfeiffer R.M., Yang X.R. (2018). Breast cancer risk factors, survival and recurrence, and tumor molecular subtype: Analysis of 3012 women from an indigenous Asian population. Breast Cancer Res..

[6] Khalil S., Hatch L., Price C.R., Palakurty S.H., Simoneit E., Radisic A., Pargas A., Shetty I., Lyman M., Couchot P. (2019). Addressing Breast Cancer Screening Disparities Among Uninsured and Insured Patients: A Student-Run Free Clinic Initiative. J. Community Health.

[7] Clemons M., Danson S., Howell A. (2002). Tamoxifen (‘Nolvadex’): A review: Antitumour treatment. Cancer Treat. Rev..

[8] Day C.M., Hickey S.M., Song Y., Plush S.E., Garg S. (2020). Novel Tamoxifen Nanoformulations for Improving Breast Cancer Treatment: Old Wine in New Bottles. Molecules.

[9] Silvente-Poirot S., de Medina P., Record M., Poirot M. (2016). From tamoxifen to dendrogenin A: The discovery of a mammalian tumor suppressor and cholesterol metabolite. Biochimie.

[10] Wilkes G.M., Barton-Burke M. (2019). 2020–2021 Oncology Nursing Drug Handbook.

[11] Mohamed K.E., Elamin A. (2020). Adherence to endocrine therapy and its relation to disease-free survival among breast cancer patients visiting an out-patient clinic at Khartoum Oncology Hospital, Sudan. J. Eval. Clin. Pract..

[12] Riggins R.B., Schrecengost R.S., Guerrero M.S., Bouton A.H. (2007). Pathways to tamoxifen resistance. Cancer Lett..

[13] Liu S., Meng X., Chen H., Liu W., Miller T., Murph M., Lu Y., Zhang F., Gagea M., Arteaga C.L. (2014). Targeting tyrosine-kinases and estrogen receptor abrogates resistance to endocrine therapy in breast cancer. Oncotarget.

[14] Chen P., Lee N.V., Hu W., Xu M., Ferre R.A., Lam H., Bergqvist S., Solowiej J., Diehl W., He Y.-A. (2016). Spectrum and degree of CDK drug interactions predicts clinical performance. Mol. Cancer Ther..

[15] Maira S.-M., Stauffer F., Schnell C., García-Echeverría C. (2009). PI3K Inhibitors for Cancer Treatment: Where Do We Stand?.

[16] Xing C.-G., Zhu B.S., Fan X.Q., Liu H.H., Hou X., Zhao K., Qin Z.H. (2009). Effects of LY294002 on the invasiveness of human gastric cancer in vivo in nude mice. World J. Gastroenterol..

[17] Zhao W., Qiu Y., Kong D. (2017). Class I phosphatidylinositol 3-kinase inhibitors for cancer therapy. Acta Pharm. Sin. B.

[18] Rahmani F., Ferns G.A., Talebian S., Nourbakhsh M., Avan A., Shahidsales S. (2020). Role of regulatory miRNAs of the PI3K/AKT signaling pathway in the pathogenesis of breast cancer. Gene.

[19] Ortega M.A., Fraile-Martínez O., Asúnsolo Á., Buján J., García-Honduvilla N., Coca S. (2020). Signal Transduction Pathways in Breast Cancer: The Important Role of PI3K/Akt/mTOR. J. Oncol..

[20] Engelman J.A. (2009). Targeting PI3K signalling in cancer: Opportunities, challenges and limitations. Nat. Rev. Cancer.

[21] Miller L.A. (2011). The National Practitioner Data Bank: A primer for clinicians. J. Perinat. Neonatal Nurs..

[22] Falasca M. (2010). PI3K/Akt signalling pathway specific inhibitors: A novel strategy to sensitize cancer cells to anti-cancer drugs. Curr. Pharm. Des..

[23] McKenna M., McGarrigle S., Pidgeon G.P. (2018). The next generation of PI3K-Akt-mTOR pathway inhibitors in breast cancer cohorts. Biochim. Biophys. Acta Rev. Cancer.

[24] Kanaizumi H., Higashi C., Tanaka Y., Hamada M., Shinzaki W., Azumi T., Hashimoto Y., Inui H., Houjou T., Komoike Y. (2019). PI3K/Akt/mTOR signalling pathway activation in patients with ER-positive, metachronous, contralateral breast cancer treated with hormone therapy. Oncol. Lett..

[25] Li D., Ji H., Niu X., Yin L., Wang Y., Gu Y., Wang J., Zhou X., Zhang H., Zhang Q. (2020). Tumor-associated macrophages secrete CC-chemokine ligand 2 and induce tamoxifen resistance by activating PI3K/Akt/mTOR in breast cancer. Cancer Sci..

[26] Jagtap J.C., Parveen D., Shah R.D., Desai A., Bhosale D., Chugh A., Ranade D., Karnik S., Khedkar B., Mathur A. (2015). Secretory prostate apoptosis response (Par)-4 sensitizes multicellular spheroids (MCS) of glioblastoma multiforme cells to tamoxifen-induced cell death. FEBS Open Bio.

[27] Ko J.C., Chiu H.C., Syu J.J., Chen C.Y., Jian Y.T., Huang Y.J., Wo T.Y., Jian Y.J., Chang P.Y., Wang T.J. (2015). Down-regulation of MSH2 expression by Hsp90 inhibition enhances cytotoxicity affected by tamoxifen in human lung cancer cells. Biochem. Biophys. Res. Commun..

[28] Ko J.C., Chiu H.C., Syu J.J., Jian Y.J., Chen C.Y., Jian Y.T., Huang Y.J., Wo T.Y., Lin Y.W. (2014). Tamoxifen enhances erlotinib-induced cytotoxicity through down-regulating AKT-mediated thymidine phosphorylase expression in human non-small-cell lung cancer cells. Biochem. Pharmacol..

[29] Li C., Zhou C., Wang S., Feng Y., Lin W., Lin S., Wang Y., Huang H., Liu P., Mu Y.-G. (2011). Sensitization of glioma cells to tamoxifen-induced apoptosis by Pl3-kinase inhibitor through the GSK-3beta/beta-catenin signaling pathway. PLoS ONE.

[30] Pu X., Storr S.J., Zhang Y., Rakha E.A., Green A.R., Ellis I.O., Martin S.G. (2017). Caspase-3 and caspase-8 expression in breast cancer: Caspase-3 is associated with survival. Apoptosis.

[31] Nassar A., Lawson D., Cotsonis G., Cohen C. (2008). Survivin and caspase-3 expression in breast cancer: Correlation with prognostic parameters, proliferation, angiogenesis, and outcome. Appl. Immunohistochem. Mol. Morphol..

[32] Cui Q., Yu J.H., Wu J.N., Tashiro S.I., Onodera S., Minami M., Ikejima T. (2007). P53-mediated cell cycle arrest and apoptosis through a caspase-3- independent, but caspase-9-dependent pathway in oridonin-treated MCF-7 human breast cancer cells. Acta Pharmacol. Sin..

[33] O’Donovan N., Crown J., Stunell H., Hill A.D., McDermott E., O’Higgins N., Duffy M.J. (2003). Caspase 3 in breast cancer. Clin. Cancer Res..

[34] Abbas T., Dutta A. (2009). p21 in cancer: Intricate networks and multiple activities. Nat. Rev. Cancer.

[35] Thomadaki H., Scorilas A. (2008). Molecular profile of the BCL2 family of the apoptosis related genes in breast cancer cells after treatment with cytotoxic/cytostatic drugs. Connect. Tissue Res..

[36] Luna-Vargas M.P.A., Chipuk J.E. (2016). Physiological and Pharmacological Control of BAK, BAX, and Beyond. Trends Cell Biol..

[37] Da Veiga G.L., da Silva RD M., Pereira E.C., Azzalis L.A., da Costa Aguiar Alves B., de Sousa Gehrke F., Gascón T.M., Fonseca F.L.A. (2019). The role of Survivin as a biomarker and potential prognostic factor for breast cancer. Rev. Assoc. Med. Bras..

[38] Motawi T.M.K., Zakhary N.I., Darwish H.A., Abdalla H.M., Tadros S.A. (2019). Significance of Serum Survivin and -31G/C Gene Polymorphism in the Early Diagnosis of Breast Cancer in Egypt. Clin. Breast Cancer.

[39] Feng F.F., Zhang D.R., Tian K.L., Lou H.Y., Qi X.L., Wang Y.C., Duan C.X., Jia L.J., Wang F.H., Liu Y. (2011). Growth inhibition and induction of apoptosis in MCF-7 breast cancer cells by oridonin nanosuspension. Drug Deliv..

[40] Kumar A., D’Souza S.S., Gaonkar S.L., Rai K.L., Salimath B.P. (2008). Growth inhibition and induction of apoptosis in MCF-7 breast cancer cells by a new series of substituted-1,3,4-oxadiazole derivatives. Investig. New Drugs.

[41] Jänicke R.U., Sprengart M.L., Wati M.R., Porter A.G. (1998). Caspase-3 is required for DNA fragmentation and morphological changes associated with apoptosis. J. Biol. Chem..

[42] Jiang H., Fan D., Zhou G., Li X., Deng H. (2010). Phosphatidylinositol 3-kinase inhibitor(LY294002) induces apoptosis of human nasopharyngeal carcinoma in vitro and in vivo. J. Exp. Clin. Cancer Res..

[43] Post A.E.M., Bussink J., Sweep F.C., Span P.N. (2020). Changes in DNA Damage Repair Gene Expression and Cell Cycle Gene Expression Do Not Explain Radioresistance in Tamoxifen-Resistant Breast Cancer. Oncol. Res..

[44] Andre F., Nahta R., Conforti R., Boulet T., Aziz M., Yuan LX H., Meslin F., Spielmann M., Tomasic G., Pusztai L. (2008). Expression patterns and predictive value of phosphorylated AKT in early-stage breast cancer. Ann. Oncol..

[45] Yang Z.Y., Di M.Y., Yuan J.Q., Shen W.X., Zheng D.Y., Chen J.Z., Mao C., Tang J.L. (2015). The prognostic value of phosphorylated Akt in breast cancer: A systematic review. Sci. Rep..

[46] Alkahtani H.M., Abdalla A.N., Obaidullah A.J., Alanazi M.M., Almehizia A.A., Alanazi M.G., Ahmed A.Y., Alwassil O.I., Darwish H.W., Abdel-Aziz A.A.-M. (2020). Synthesis, cytotoxic evaluation, and molecular docking studies of novel quinazoline derivatives with benzenesulfonamide and anilide tails: Dual inhibitors of EGFR/HER2. Bioorg. Chem..

[47] Gouda A.M., Abdelazeem A.H., Abdalla A.N., Ahmed M. (2018). Pyrrolizine-5-carboxamides: Exploring the impact of various substituents on anti-inflammatory and anticancer activities. Acta Pharm..

[48] Fall A.D., Bagla VP B., Bassene E., Eloff J.N. (2017). Phytochemical Screening, Antimicrobial and Cytotoxicity Studies of Ethanol Leaf Extract of Aphaniasenegalensis (Sapindaceae). Afr. J. Tradit. Complement. Altern. Med..

[49] Abdalla A.N., Shaheen U., Abdallah Q., Flamini G., Bkhaitan M.M., Abdelhady M.I., Ascrizzi R., Bader A. (2020). Proapoptotic Activity of Achillea membranacea Essential Oil and Its Major Constituent 1,8-Cineole against A2780 Ovarian Cancer Cells. Molecules.

[50] Malki W.H., Gouda A.M., Ali H.E., Al-Rousan R., Samaha D., Abdalla A.N., Bustamante J., Elmageed Z.Y.A., Ali H.I. (2018). Structural-based design, synthesis, and antitumor activity of novel alloxazine analogues with potential selective kinase inhibition. Eur. J. Med. Chem..

[51] Abdalla A.N., Abdallah M.E., Aslam A., Bader A., Vassallo A., De Tommasi N., Malki W.H., Gouda A.M., Mukhtar M.H., El-Readi M.Z. (2020). Synergistic Anti Leukemia Effect of a Novel Hsp90 and a Pan Cyclin Dependent Kinase Inhibitors. Molecules.

[52] Shaheen U., Ragab E.A., Abdalla A.N., Bader A. (2018). Triterpenoidal saponins from the fruits of Gleditsia caspica with proapoptotic properties. Phytochemistry.

